# Quantitative intrinsic auto-cathodoluminescence can resolve spectral signatures of tissue-isolated collagen extracellular matrix

**DOI:** 10.1038/s42003-019-0313-x

**Published:** 2019-02-18

**Authors:** Marcin S. Zielinski, Elif Vardar, Ganesh Vythilingam, Eva-Maria Engelhardt, Jeffrey A. Hubbell, Peter Frey, Hans M. Larsson

**Affiliations:** 1 Attolight AG, Lausanne, 1015 Switzerland; 2 Institute for Bioengineering, School of Life Sciences and School of Engineering, École Polytechnique Fédérale de Lausanne, Lausanne, 1015 Switzerland; 3 Department of Pediatrics, Centre Hospitalier Universitaire Vaudois (CHUV), Lausanne, 1011 Switzerland; 4 Department of Surgery, Faculty of Medicine, University Malaya, Kuala Lumpur, 53100 Malaysia; 5 Institute for Molecular Engineering, University of Chicago, Chicago, IL 60637 USA

## Abstract

By analyzing isolated collagen gel samples, we demonstrated in situ detection of spectrally deconvoluted auto-cathodoluminescence signatures of specific molecular content with precise spatial localization over a maximum field of view of 300 µm. Correlation of the secondary electron and the hyperspectral images proved ~40 nm resolution in the optical channel, obtained due to a short carrier diffusion length, suppressed by fibril dimensions and poor electrical conductivity specific to their organic composition. By correlating spectrally analyzed auto-cathodoluminescence with mass spectroscopy data, we differentiated spectral signatures of two extracellular matrices, namely human fibrin complex and rat tail collagen isolate, and uncovered differences in protein distributions of isolated extracellular matrix networks of heterogeneous populations. Furthermore, we demonstrated that cathodoluminescence can monitor the progress of a human cell-mediated remodeling process, where human collagenous matrix was deposited within a rat collagenous matrix. The revealed change of the heterogeneous biological composition was confirmed by mass spectroscopy.

## Abstract (web summary)

Zielinski et al. show that quantitative label-free cathodoluminescence-scanning electron microscopy differentiates spectral signatures of two extracellular matrices. This method can monitor the progress of a smooth muscle cell-mediated remodeling process without using antibodies to enhance the optical signal.

## Introduction

An ability to identify structural features with the spatial resolution of an electron microscope (EM) with simultaneous detection of molecular composition by utilizing only the optical properties of a studied material would allow a deeper understanding of complex heterogeneous biological structures. Recent milestones in the field of cryo-electron microscopy of complex bioorganic structures have delivered resolution close to that of a single atom, with fast integration rates allowing single protein imaging. Correlation of cryo-electron microscopy image contrast with molecular modeling has unveiled, e.g., conformation structures and dynamics of isolated proteins^1–3^, receptors controlling ionic fluxes through a membrane pore channel^4^, and brain filaments involved in neurodegenerative disease^5^. Isolated protein structural details at a single-molecule level were also recently spatially resolved by a low-energy electron holography technique^6^. Furthermore, larger scale objects, e.g., artificial self-assembled and biomineralized peptide-amphiphile fibers have been investigated^7^, showing the potential of electron microscopy in characterization of complex organic scaffolds and molecular scale processes in tissue engineering.

When the studied material is not an isolated molecular structure, but a network of proteins, such as a multi-component collagen fibril, obtaining structural and compositional information requires correlative techniques combining electron microscopy with, e.g., X-ray crystallography and molecular modeling to fully understand structural features at a single fiber–fibril level^8,9^. Resolving a complex fibrillar tissue structure in situ, however, becomes extremely challenging since different types of cross-linked collagens and interacting proteins might be involved. In this case, typical characterization techniques, such as histology, immunostaining^10^, scanning electron microscopy (SEM) or diffraction-limited optical methods based on fluorescence or second-harmonic generation (SHG)^11–13^ cannot provide unambiguous compositional information. Recent examples of correlating SEM with energy-dispersive X-ray spectroscopy (EDS), showed characterization of critical insights in pathological processes in calcified lesions in cardiovascular tissues^14^ and helped to identify typical amino-acid fragments of collagen fibrils in preserved prehistoric specimens^15^.

Another technique correlates with cathodoluminescence (CL) image contrast, which has a similar physical origin as photoluminescence^16^. In case of an organic molecular system, cathodoluminescence results from excitation of higher vibrational molecular states under exposure to an incident electron beam, proceeded by the internal conversion to *S*_1_ and return to the ground state *S*_0_, which can be assisted with a cathodoluminescence photon emission. Unlike fluorescence microscopy, because the incident electron beam energy is within a 10^3^ eV range, there is no need to match the incident wavelength with the absorption spectra of the studied material. As a consequence, even the wide band-gap materials can be excited, and cathodoluminescence photons occur among other elastic or inelastic interactions of an incident electron and the exposed material, which can lead to a variety of different contrasts^17^. Additionally, cathodoluminescence can also provide spectral information about a studied material^18,19^, alternative to that of photoluminescence utilized in a regular optical microscopy. Cathodoluminescence imaging of biological samples, however, is extremely challenging due to technological limitations in efficient collection of usually very low intensity optical signals from biological specimens. For this reason, reports of cathodoluminescence on bioimaging usually take advantage either of staining with bright nanolabels^20,21^, or correlating backscattered electron (BE) and low-resolution cathodoluminescence images generated through a SiN membrane with a probe size of a few tens of nm. Such an example was reported by Nawa et al., demonstrating dynamic auto-cathodoluminescence (auto-CL) imaging of HeLa cells, with light collection through standard optical components in transmission geometry and without spectral analysis^22^.

**Fig. 1:**
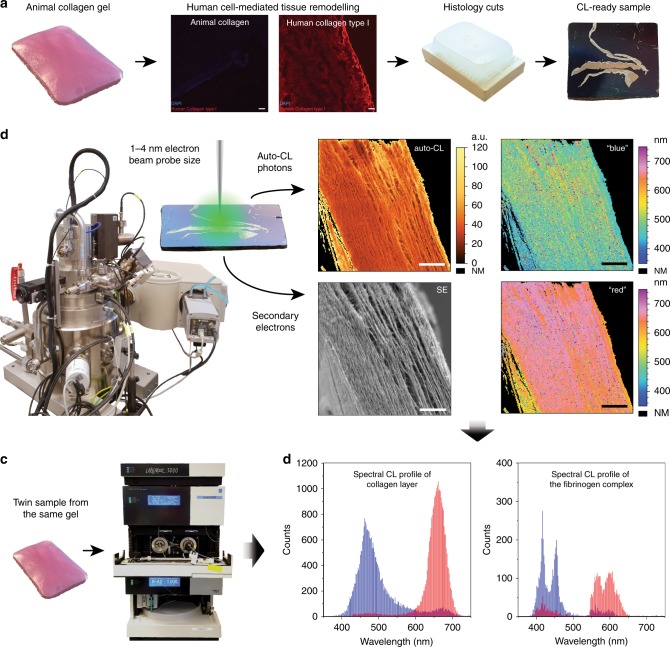
Schematic workflow in CL-SEM characterization of animal and humanized collagen gels. **a** Preparation of animal/human collagen tissues for CL-SEM analysis. Samples were prepared before/after human cell-mediated remodeling as 80 µm histology cuts, then transferred onto a silicone substrate, followed by paraffin removal, critical drying, and coating with 4 nm thin layer of Au–Pd. **b** Hyperspectral CL-SEM characterization of collagen tissues, providing secondary electron surface topography image and hyperspectral auto-CL map of the corresponding area. Obtained hyperspectral CL data is deconvoluted into “blue” and “red” components and spatially resolved; scale bars are 25 µm. Detailed scheme of data analysis is provided in the Supplementary Fig. 2. **c** Mass spectroscopy analysis of a twin sample obtained from the same collagen gel. **d** Spectral auto-CL distributions of the two deconvoluted components. Final data interpretation is based on correlation with mass spectroscopy and CL reference data

In this manuscript, we demonstrate the application of a highly sensitive quantitative CL-SEM microscope, capable of detecting an intrinsic auto-cathodoluminescence signal from a soft collagen matrix when exposed to a ~4 nm sized probe. We show that spectrally resolved cathodoluminescence microscopy can unveil material composition and its spatial distribution in extracellular matrices of collagen isolated from bovine and rat sources, without using antibodies, nanolabels, or fluorophores to enhance the optical signal. This capability appears to provide a useful characterization method, especially in the case when no specific antibodies are available, and when characterization of structural properties of complex biological systems is challenging. We suggest a pathway toward high resolution electron microscopy imaging, correlated with spectrally resolved and deconvoluted intrinsic cathodoluminescence information and mass spectroscopy data, to provide a new label-free integrative technique in compositional and spatial characterization of complex heterogenous biomaterials. The schematic workflow of this study is shown in Fig. 1.

## Results

### Spectrally resolved auto-CL from individual collagen fibrils

We examined several types of collagen-based samples using a dedicated CL-SEM microscope Attolight Rosa 4634 and data processing method, described in more detail in the Supplementary Figs. 1, 2, respectively. This microscope has been originally designed for cathodoluminescence characterization of a broad range of inorganic^23–26^ and rarely organometallic^27^ materials. Here, we present proof of principle results on organic biological material samples with such a high cathodoluminescence detection capacity microscope.

**Fig. 2:**
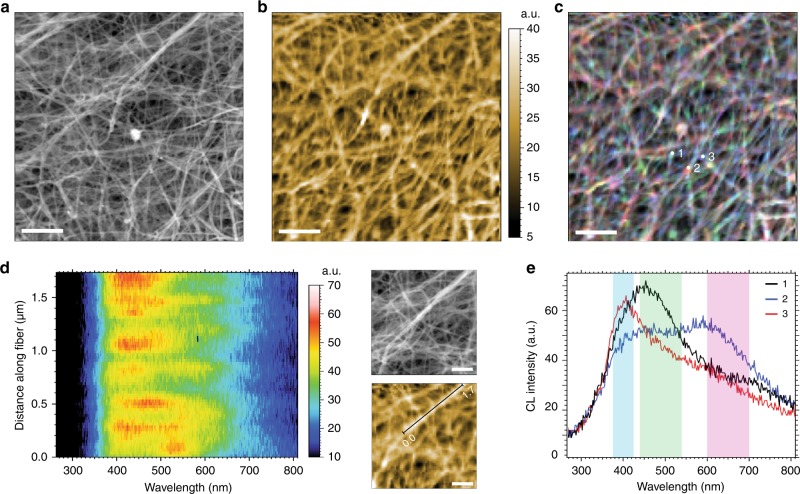
High resolution SEM and spectrally resolved intrinsic auto-cathodoluminescence images of a bovine collagen gel. **a** SE image (5.55 µm FOV, 10.8 nm pixel size), and **b** corresponding hyperspectral auto-CL image (43.2 nm pixel size, 550 nm spectral bandwidth, 20 ms pixel integration time). The bright particle in the center is residual gold remaining after sputtering, which emits CL above 800 nm, out of the detection range. **c** An overlay of three monochromatic auto-CL images, where selected blue, green, and red CL bands, marked in **e**, are centered at: 400 nm (50 nm bandwidth), 490 nm (100 nm bandwidth), and 650 nm (100 nm bandwidth), respectively. **d** Spectra projection showing auto-CL emission along a thick collagen fibril (1.73 µm distance, marked in the zoomed auto-CL image); each spectrum corresponds to a single pixel within the CL image (43.2 nm distance along the fibril). **e** Point spectra extracted from isolated fibrils and intersections of fibrils, showing CL spectral information specific to a molecular content. Scale bars in **a**, **b** and **c** are 2 µm. Scale bars in **d** are 500 nm

First, we examined the surface morphology of a low fiber density bulky bovine collagen sample, showing that secondary electron (SE) contrast revealed nanometer sized isolated extracellular fibrils in the analyzed collagen (Fig. 2a). The sample was prepared by first sectioning to create a flat imaging interface and then sputtering with a 4 nm thick Au–Pd layer to provide an electrical contact, as detailed in the methods section. Highly efficient optical collection allowed performance of label-free auto-cathodoluminescence hyperspectral imaging correlated with the secondary electron contrast with a small sample drift involved. The detected intrinsic auto-cathodoluminescence signal in Fig. 2b, c revealed complex spectral information from individual fibers and their entangled networks with high spatial precision. No image drift correction was applied to the presented result, yet correlation of individual fibers on the secondary electron and cathodoluminescence images was possible despite a slight sample drift. To our best knowledge, these label-free auto-cathodoluminescence measurements show for the first time nanometer-scale image resolution in a complex biological sample network. In this particular case, the cathodoluminescence hyperspectral map was recorded with 43.2 nm pixel size, achieved by focusing a ~4 nm tight beam probe at 8 keV energy on individual collagen fibrils. This pixel size has been intentionally set to compromise 20 ms cathodoluminescence integration time, providing sufficient signal-to-noise ratio (SNR) over a 550 nm spectral bandwidth within a single pixel. The SNR, however, can be improved by adapting the microscope diffraction and detection components specifically for this application. While the spatial resolution in the secondary electron contrast can easily reach 2–3 nm, the cathodoluminescence image resolution is first determined by a single fibril dimension and poor conductivity specific to its organic content. Since generated charge carriers cannot diffuse over a long distance within the organic material, the radiative recombination occurs within an area limited either by the excitation volume (beam energy dependent), or local dimension of the exposed material and its cross-section contributing into the radiative process. Our experimental results suggest that with the further microscope improvement, the maximum cathodoluminescence resolution could potentially reach <30 nm.

Interestingly, despite irradiation with 8 keV beam energy, expected to result in broad and deep excitation volumes, the panchromatic cathodoluminescence image (Fig. 2b) revealed most of the optical information from the top layer collagen fibrils. Therefore, most of the generated cathodoluminescence signal results from absorption of the incident beam energy by directly irradiated material, vibrational energy relaxation and internal conversion into the *S*_1_ molecular singlet state from where a cathodoluminescence photon emission occurs, as suggested by Fisher et al.^16^. Because of the low signal level observed from deeper collagen layers in the background, we estimate that a maximum of 5–10% of the generated cathodoluminescence emission originates from reabsorption of emitted secondary electrons or X-rays.

A spectrally resolved cathodoluminescence image of bovine collagen is presented in Fig. 2c as an overlay of three monochromatic auto-cathodoluminescence emission bands, marked in Fig. 2e as: blue—centered at 400 nm (50 nm bandwidth), green—centered at 490 nm (100 nm bandwidth), and red—centered at 650 nm (100 nm bandwidth). These three bands were selected to highlight spatial localization of the detected cathodoluminescence emission peaks, shown in the point spectra (Fig. 2e), to reveal a heterogeneous molecular composition of the material. Examining a specific collagen fibril within a 1.73 µm distance, as seen in Fig. 2d, provided a spectrally resolved projection with a color-coded cathodoluminescence signal intensity. Each pixel row in this profile represents a single auto-cathodoluminescence spectrum, obtained by probing every 43.2 nm along the selected fibril. First, this proves very short carrier diffusion lengths in this type of bioorganic material. Second, broadband spectral information dependent on the molecular composition can be obtained with a high spatial precision, and closer examination of single fibrils and their junctions by means of auto-cathodoluminescence can identify a diverse pattern of molecular species from the isolated bovine collagen.

We confirmed by mass spectrometry that the bovine collagen used is a heterogeneous extracellular matrix (ECM) contains a complex molecular composition of seven proteins (Supplementary Fig. 3a,b and Supplementary Table 1). Clearly, the majority is bovine type I collagen consisting of isoform alpha-1 and -2, however, also minor amounts of five other bovine ECM proteins and blood proteins were identified: collagen alpha-1(III), collagen alpha-1(II), collagen alpha-1(IV), lumican, and serum albumin.

One could expect the observed cathodoluminescence signal to originate from the Au–Pd coating layer, in which the presence of multiple propagating plasmon eigenmodes appear as random hot spots with enhanced field strength and confinement, as reported in complex metallic networks^28–30^. Figure 2c clearly shows that the observed cathodoluminescence signal completely follows the topography of collagen fibrils seen in the secondary electron image (Fig. 2a), which is in contrast to the reported metallic network behavior^30^. Additionally, no local enhancement nor plasmon coupling (hot spots) between fibrils could be found, indicating that the observed signal originates only from the intrinsic collagen auto-cathodoluminescence emission. This interpretation is additionally supported by a control cathodoluminescence experiment, where two bovine collagen samples were sputtered with 4- and 10-nm-thick Au–Pd layers and characterized using the same experimental conditions (Supplementary Fig. 4a–f). Despite differences in metal thickness, both controls resulted in very similar cathodoluminescence emission as presented in Supplementary Fig. 4c,f. Rarely observed aggregates of Au–Pd nanoparticles exhibited very bright NIR emission with a longer wavelength than the cut-off of the CCD camera (cathodoluminescence peak located at ~980 nm in the Supplementary Fig. 4g–i) and were excluded from the cathodoluminescence data analysis of all collagen samples.

### Auto-CL characterization of engineered CFC gel

**Fig. 3:**
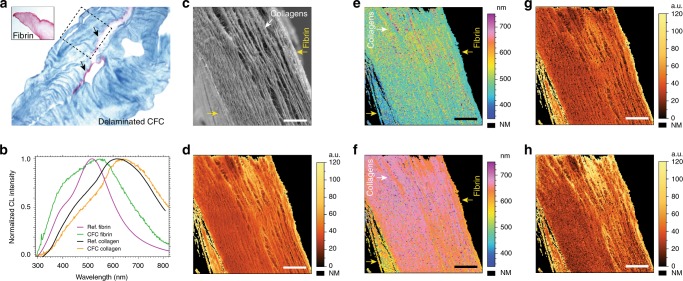
Optical histology, SEM and auto-CL images of bio-engineered hybrid rat CFC gel. **a** Masson’s trichrome (MT) stain optical image of a CFC gel with fibrin layer marked with arrows. The inset shows a MT image of an uncompressed fibrin spot. The dashed square represents an example of a delaminated region characterized with CL. **b** Normalized average auto-CL spectra of a reference fibrin sample (purple), reference rat collagen (black), rat collagen and fibrin sites of the investigated CFC sample (orange and green curves, respectively). **c** SE image of a CFC cross-sectioned sample and **d** corresponding panchromatic auto-CL image (640 nm pixel size). Spatial distribution images of the blue—shorter wavelength (**e**), and the red—longer wavelength (**f**) deconvoluted CL components. Corresponding auto-CL peak intensity maps of the blue (**g**) and the red (**h**) deconvoluted CL components. Scale bars are 25 µm, FOV = 128 µm. NM stands for non-measurable pixels, cut-off from the region of interest by intensity thresholding. Yellow arrows in the SE and CL images indicate fibrin, white arrows indicate collagens

In previously published work^31^, we have prepared hybrid fibrin and collagen gels for bladder tissue engineering purposes. These gels were made of two sheets of collagen from a rat-tail tissue source, in which a weak adhesion between these two layers was made by polymerizing a fibrin gel in between, creating a laminated collagen–fibrin–collagen (CFC) structure. The CFC sample studied in this manuscript, however, is a piece of locally delaminated gel with a single collagen layer and attached fibrin on the right-side, as marked with the dashed square in the histology (Fig. 3a). The CFC abbreviation is used similarly as in the report by Vardar et al.

To evaluate the capabilities of cathodoluminescence in characterization of different molecular content, we analyzed a CFC gel sample to see if the spectral signature of fibrin on the surface of collagen gels can be tracked and localized by utilizing only the intrinsic auto-cathodoluminescence signal. For this purpose, we performed a hyperspectral cathodoluminescence scan over a 128 µm field of view (FOV), spanning the whole width of the studied CFC sample cross-section with 200 × 200 pixels (640 nm pixel size). Obtained hyperspectral data set contains 40,000 individual cathodoluminescence spectra resulting from highly-localized spots exposed to a 4 nm large probe size of the electron beam. In Fig. 3c, d, we show that both the secondary electron and the corresponding panchromatic cathodoluminescence images clearly revealed the collagenous fibril-like structure of the matrix in the cross-section view. While the secondary electron image (Fig. 3c) could not clearly indicate a fibrin layer within the CFC gel, analysis of the cathodoluminescence data showed a blue-shifted auto-cathodoluminescence emission spectra (Fig. 3b) from the cross-section right-side edge in respect to the central collagenous part. Surprisingly, fibrin cathodoluminescence emission was also detected on the left-side facet of the studied CFC gel, visible in Fig. 3c as a side wall of the cross-section in the bottom left corner of the secondary electron image. This thin fibrin layer is present due to expansion of the fibrin excess in a mold during the CFC gel lamination process^31^. Identification of such a thin coating on a topographically-similar collagen would not be possible by means of SEM without CL.

Figure 3b shows the average normalized cathodoluminescence spectra extracted from ~26 µm^2^ areas within two zones of the sample (averaged over 64 spectra), juxtaposed with the reference cathodoluminescence spectrum measured from a pure fibrin gel sample. Similar comparison with a reference to rat collagen is presented for the collagenous part of the cathodoluminescence spectrum of the CFC sample. The slight spectral shift and difference in full width at half maximum (FWHM) between the black and orange curves arises from an increased heterogeneity of the studied sample.

In Fig. 3b, the average cathodoluminescence spectrum detected from fibrin sites (green curve) shows a similar shape and matching ~400 nm peak position with the reference spectrum (purple curve), measured from a pure fibrin sample. The broader FWHM and ~45 nm red-shift of the right-hand peak on the CFC spectrum (green curve), suggests a more complex molecular composition of the CFC fibrin layer, most likely due to diffusion at the collagen–fibrin interface during the CFC preparation process. The average cathodoluminescence spectrum from collagen, on the other hand, exhibits different symmetry and a dominant peak located at ~655 nm, allowing for easy distinction of the collagen layer.

In the next step, each of the 40,000 spectra of the hyperspectral cathodoluminescence map (Fig. 3d) was fitted using a combination of pseudo-Voigt functions and deconvoluted into two components. For clarity, we call these components blue and red, which correspond to a shorter and longer wavelength peak distribution range of fitted spectra, respectively. Choosing two deconvolution components simplified the data analysis process, yet still remains in agreement with point spectra observed at different regions of the CFC sample. As such, either one or two clearly distinguishable peaks within the 350–750 nm range are revealed. It is clear though, that cathodoluminescence spectra exhibiting broader FWHM should be analyzed with a larger number of deconvolution components, especially when a high complexity of a biomaterial composition is expressed. More complex analysis of high resolution spectral data would potentially unveil more specific information about molecular content and interactions.

In this study, however, we focused on demonstrating the capability of spatial differentiation and characterization of two spectrally-different layers to see if cathodoluminescence can determine their heterogeneity. Spatial distributions of the two deconvoluted component peaks are presented in Fig. 3e, f, and they correlate well with the average cathodoluminescence spectra in Fig. 3b. When comparing with the central collagenous part, one can clearly identify two regions with shorter wavelengths in both components on the borders of the cross-section characteristic of the fibrin-like molecular content. Therefore, spectral cathodoluminescence characterization confirmed the CFC sample to be a region of delaminated collagen scaffold with a single collagen section and the main fibrin layer, marked by the arrow on the right edge in Fig. 3a. The thin layer of fibrin residue deposited on the opposite collagen side was also clearly identified. These two fibrin borders exhibit brighter cathodoluminescence emission intensities as shown in the panchromatic cathodoluminescence image (Fig. 3d) and in the deconvoluted component intensity maps (Fig. 3g, h). This suggests either higher intrinsic cathodoluminescence efficiency of the corresponding molecular material, or most likely a higher material packing density within the excitation volume.

In a further step, we use descriptive frequency histograms obtained directly from spatial distribution maps of the deconvoluted cathodoluminescence peaks (Fig. 3e, f) to show discrete populations of cathodoluminescence peak wavelengths of both deconvoluted components, which we call spectral signatures for simplicity. These discrete population peaks are further correlated with mass spectroscopy results and reference cathodoluminescence data in an attempt to assign specific population peak wavelengths in histograms to an adequate molecular content.

**Fig. 4:**
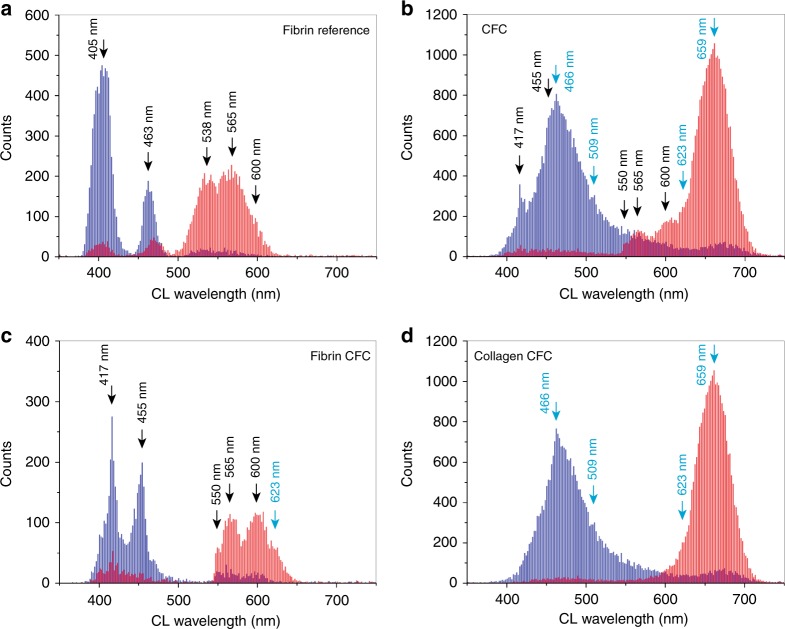
Spectral distribution analysis of deconvoluted spectral components of the CFC and reference fibrin hyperspectral CL data. **a** Deconvoluted CL histogram of the fibrin reference sample, showing distribution of the blue and red components, corresponding to shorter and longer wavelength CL emission peaks, respectively. **b** Deconvoluted CL histogram of the CFC gel; fibrin spectral signatures are marked by arrows. **c** Deconvoluted CL histogram of the separated fibrin zone from the CFC sample. **d** Deconvoluted CL histogram of the collagen zone within the CFC gel. Black labels refer to fibrin signatures, light-blue labels refer to collagen signatures

Spectral distribution histograms of the CFC gel and fibrin reference samples are presented in Fig. 4. The fibrin reference histogram (Fig. 4a) exhibits two characteristic peak signatures in the blue deconvolution component at 405 and 463 nm, and two clear peak signatures in the red component, centered at 538 and 565 nm, among a shoulder peak at 600 nm. Groups of low-count signature populations of two bands, e.g., red 405 nm, resulted from broad single peak emission spectra fitted with the same wavelength value for both deconvolution components. These replicas were excluded from the data interpretation.

Similar fibrin spectral signatures, marked by black arrows in Fig. 4b, are present in the characterized CFC gel histogram. Separate spectral distribution of CFC fibrin layers (Fig. 4c), revealed slight differences in peak distributions of both spectral components in respect to the reference fibrin histogram (Fig. 4a), showing narrower FWHM and 8 and 35 nm cathodoluminescence emission peak shifts of the blue component signatures. Interestingly, the highly expressed 538 nm reference fibrin signature of the red component was not observed in the CFC sample, where instead a low-count signature at 550 nm is present among the 565 and 600 nm signature peaks. The CFC red component shows an additional signature at 623 nm (light-blue label in Fig. 4c), suggesting either different protein ratios within the fibrin layer or the presence of incorporated collagen fibers within the fibrin layer.

**Fig. 5:**
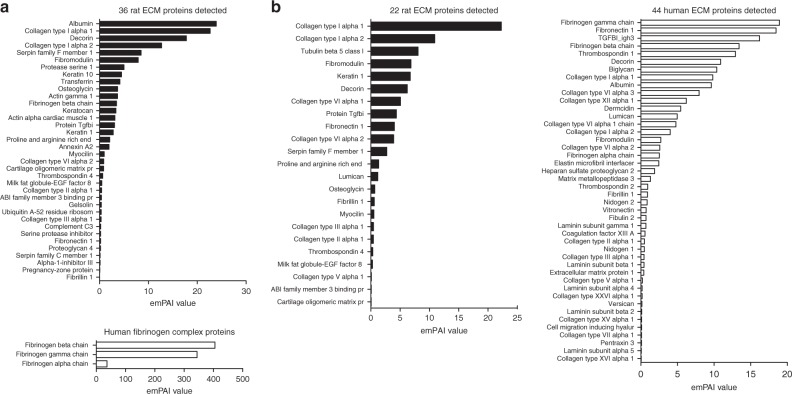
Mass spectra of expressed ECM proteins in the bio-engineered collagen gels. **a** CFC gel sample with 36 rat proteins detected using DAVID online software and extracellular space and region as a selection GO-term, with an additional three human fibrinogen proteins detected using fibrinogen complex as GO-term^35,36^. **b** Four weeks humanized-CFC cell-free gel, with 44 human ECM and 22 rat ECM proteins detected using extracellular space and region as a selection GO-term

In contrast, a histogram of the collagenous part of the CFC sample in Fig. 4d (without selecting fibrin layers), shows two main broad peak signatures of both deconvoluted components centered at 466 and 659 nm among two less visible peaks at 509 and 623 nm. In the complete CFC histogram (collagen and fibrin, Fig. 4b), the 466 nm peak signature overshadows the 455 nm fibrin signature. In the corresponding secondary electron image (Fig. 3c), the collagen layer exhibits some morphological differences between individual fibril structures, and this correlates with the deconvoluted cathodoluminescence images (Fig. 3e, f), with clearly resolved lines of spectrally shifted cathodoluminescence emission along different fibrils. These structural and compositional features were detected with a 4 nm large electron beam probe irradiating isolated collagen fibers over a 128 µm FOV every 640 nm, providing ~26,800 sets of deconvoluted cathodoluminescence spectra of the CFC collagen (assuming one third of the cathodoluminescence image in Fig. 3d, without the black area of non-measurable points marked as NM). In the spectral distribution collagen analysis (Fig. 4d), FWHM of the red and the blue deconvoluted components is 59 and 47 nm, respectively, which suggests more complex molecular composition than the ~11–16 nm FWHM broad blue fibrin signatures in Fig. 4c. This observation is supported by mass-spectrometry-resolved proteome of the analyzed CFC material (Fig. 5a), showing that 36 ECM rat proteins were detected along with three proteins of the human fibrinogen complex. In total, 62 rat proteins were detected and quantified (Supplementary Table 2) using an exponential modified protein abundance index (emPAI)^32^.

### CL characterization of smooth muscle cell-remodeled CFC gels

Humanized-CFC gels were prepared by adding human smooth muscle cells (hSMC) into the collagen solution prior to the gelation phase. Cells were cultured for 2 and 4 weeks in CFC gels after which a decellularization treatment was done to result in cell-free humanized-CFC gels. Immunohistochemistry with a collagen type I antibody specific for human and not rat, revealed that ECM remodeling by the human cells is gradual, when analyzing periods of 1, 2, and 4 weeks of cultured human cells in the laminated rat collagen gels (Supplementary Fig. 5). After 2 and 4 weeks of culture, the hSMC cells were removed using a decellularization protocol^33^, and confirmation that the cellular components were removed was performed using double-stranded DNA (dsDNA) analysis. The DNA content was below 50 ng dsDNA mg^−1^ of matrix, a set limit for successful decellularization (Supplementary Fig. 6)^34^, thereby creating the decellularized humanized-CFC extracellular matrix gels, hereafter referred to 2 weeks cell-free and 4 weeks cell-free gels.

**Fig. 6:**
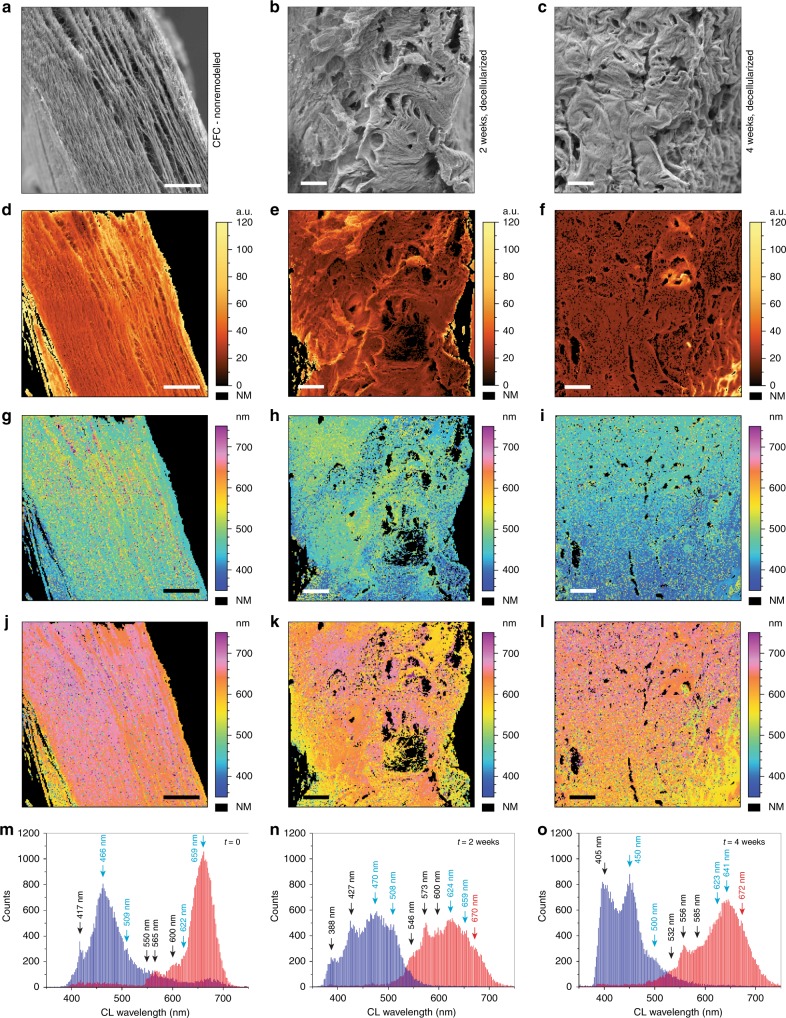
Characterization of hSMC cell-remodeled humanized collagen gels. **a**–**c** SE images of the CFC, 2 and 4 weeks cell-free gels, respectively. **d**–**f** Panchromatic auto-CL images of the CFC, 2 and 4 weeks cell-free gel samples, respectively. **g**–**i** Corresponding distribution images of the blue deconvoluted component, and **j**–**l** the red deconvoluted component. **m**–**o** Corresponding deconvoluted CL histograms. The CFC data is shown as the reference and starting point of the remodeling process. Scale bars are 25 µm, and the FOV in **b**, **c** is 180 µm; each auto-CL hyperspectral scan was obtained with 200 × 200 pixels. Black labels refer to human fibrinogen complex signatures, light-blue labels refer to collagen signatures, and red labels indicate CL signatures from unidentified molecular content

Initial SEM characterization of hSMC cell-remodeled extracellular matrixes (Fig. 6b, c) revealed a denser architecture with more randomized fibril arrangement that increased with culture time. This trend was also observed in the panchromatic auto-cathodoluminescence contrast images (Fig. 6d–f). Cross-sections of both 2 and 4 weeks cell-free gels were analyzed by means of spectral deconvolution (Fig. 6h, i, k, l, and Supplementary Fig. 7), using exactly the same procedure as described above for the CFC gel. The CFC data is shown in Fig. 6g, j as a reference, and a starting point before the humanization process. Among the three analyzed samples, the 2 weeks cell-free gel images (Fig. 6h, k) show the most randomized spatial distribution of deconvoluted components. Broad areas of shorter wavelength cathodoluminescence peaks corresponding to fibrin-like signatures appearing as bluish (Fig. 6h) and greenish (Fig. 6k) color-coded pixels are visible, among highly fragmented regions of longer wavelength cathodoluminescence peaks of collagen-like signatures (particularly in the Fig. 6k). These highly fragmented distributions suggest that after 2 weeks, the hSMC cell-remodeling process was not complete. After two additional weeks of cell culture, deconvoluted images (Fig. 6i, l) appear to be more homogenous, yet small spots of varied cathodoluminescence emission peaks can be still distinguished. Additionally, a gradient of spectral blue-shift toward the right bottom corner can be observed in both components, suggesting that the cell-remodeling process was still in progress. This was confirmed by mass spectroscopy (Fig. 5b), where 22 ECM rat proteins were detected among 44 human ECM proteins. These data prove that spectrally resolved deconvoluted auto-cathodoluminescence images can provide clear information about compositional uniformity of bio-engineered tissue samples with high precision in the localization of different biomolecular materials (Fig. 2c). The corresponding spectral distribution histograms of both cell-remodeled gels (Fig. 6n, o) confirm increased compositional diversity. Both maps show that certain amount of collagen signatures (light-blue labels) were diminished, and the overall distribution of both deconvoluted components, including fibrin spectral signatures, became broader when compared with the initial CFC sample (Fig. 6m). It is difficult to precisely assign an expressed protein detected by mass spectroscopy (Fig. 5b) to a particular spectral cathodoluminescence signature; this would require prior cathodoluminescence characterization of each of the isolated pure proteins. This proof of concept study demonstrates that spectrally deconvoluted auto-cathodoluminescence data strongly correlates with the obtained mass spectra. In particular, fibrin associated proteins in the 4 weeks cell-free gel, where high contents of human fibrinogen gamma and beta chains, as well as human fibronectin-1 and fibrinogen alpha chains were detected (Fig. 5b). After 2 weeks of cell-mediated remodeling and decellularization, the blue 417 nm CFC fibrin signature was replaced by two peaks at 388 and 427 nm (Fig. 6n), which further evolved into a highly expressed broader peak signature centered at 405 nm (Fig. 6o), overlapping with the 450 nm signature marked as collagenous material. In the red deconvolution component, after 2 weeks the initial CFC fibrin peak signatures at 550, 565, and 600 nm exhibit a 4-8 nm spectral shift with a much higher expression rate. After 4 weeks of cell-mediated remodeling, these signatures shifted up to 18 nm, but their mutual ratio remained similar for each sample. Detected spectral shifts and high expression of the 405 nm blue signature (most likely overshadowing diminished 388 and 427 nm peaks), could therefore be correlated with a change of a molecular content of the human fibrinogen complex/fibrin. In particular, the appearance of human fibronectin-1, which is known to be produced by the hSMC cells^37^, was not present in the initial CFC matrix, but was later observed in the 4 weeks cell-free gel as shown in the mass-spectrometry data (Fig. 5). This protein is reported to bind to fibrinogen/fibrin^38,39^ and collagen^40^, which may explain the spread of the ~405 nm spectral signature in Fig. 6h–j within the inner part of the matrix.

Analysis of the collagen cathodoluminescence signatures in the 2 weeks cell-free gel showed much lower expression rates of the ~470 and 659 nm peaks, with a ~4 nm red-shift of the first peak, possibly related to fibronectin-1 binding to collagen. On the contrary, the 4 weeks cell-free gel revealed an increase and spectral shifts of ~450 and 641 nm collagen cathodoluminescence signatures, suggesting deposition of human collagen and its interaction with fibronectin-1^40^. Interestingly, the 2 and 4 weeks cell-free gel samples show an additional red component signature around 670 nm (labeled in red), which could be related to a high quantity of the cell-produced fibronectin-1, transforming growth factor BI ig_h3, decorin and/or biglycan detected in the 4 weeks gel mass spectrum (Fig. 5b). All of these 4 ECM or ECM-associated proteins are known to interact with collagen type I^40–42^, and could be a potential source of this signal.

One should also consider the diameters of interacting ECM protein fibers, resulting in thicker fibril structures, e.g., ~1.5 nm diameter collagen type I fiber possibly interacting with ~8 nm TGF-β^43,44^, or one of the fibrinogen complex fiber (~4.5 nm) proteins, e.g., fibronectin (~3 nm)^45,46^. Such a complex fibril, thicker than the electron probe size but still smaller than a carrier diffusion length, would strongly limit spectral identification of a single building block of fibers within by shifting and/or broadening of spectral signatures. We cannot exclude that a peak shift can also be due to the rise of human collagen type I produced by the cells, as confirmed by collagen type I antibody staining and mass spectra (Fig. 5b, and Supplementary Table 3). This was shown to be due to collagen type 1 alpha 1 isoform which exhibits a slight difference in amino-acid sequence, using a pairwise protein alignment of rat and human isoforms (92.8% pairwise alignment of COL1A1).

Finally, comparison of the initial CFC collagen signature peak at 659 nm with the 4 weeks cell-free gel collagen signature at 641 nm indicates nearly 100% of the initial amount of collagen being expressed in the remodeled tissue sample (assuming only ~66% of the image in Fig. 6j as collagen area), which still comprises a certain amount of rat material. This correlates with the mass spectroscopy results (Fig. 5b), showing heterogenous composition of 44 human ECM proteins detected among 22 ECM of rat proteins, which gives about ~66% human cell ECM remodeling efficiency.

### Conclusions

In summary, we have presented the capability of spectrally resolved cathodoluminescence microscopy in the identification and localization of ECM protein complexes in bio-engineered soft biological samples by detecting only the intrinsic emission. To our best knowledge, this study is the first demonstration of spectrally resolved label-free auto-cathodoluminescence imaging in a complex biomaterial sample. Due to the 4 nm electron beam probe size and efficient light collection, highly-localized luminescence from isolated fibrils was able to be detected with a high spatial resolution, providing an insight into the material composition of rat and bovine collagen matrices. Moreover, presented aberration-free hyperspectral images were obtained over a 180 µm FOV (max FOV in the cathodoluminescence mode of the microscope can reach 300 µm) allowing the study of thick cross-section samples in a single scan. Cathodoluminescence information retrieved within a precision of only a few nanometers, and obtained from 40,000 points at a large macro scale, allowed for the monitoring and evaluation of cell-mediated remodeling and deposition of human material in a rat tissue matrix. Obtained deconvoluted cathodoluminescence images and spectral distribution histograms in correlation with mass-spectrometry data showed an increase in ECM protein complexity of analyzed samples, providing spatial distribution and quantitative information. This is clearly visible when comparing cathodoluminescence data of the CFC and hSMC cell-remodeled samples. Additionally, we showed that broadening of deconvoluted cathodoluminescence spectral signature peaks, as presented for bovine and rat collagens in the Supplementary Fig. 3, directly correlates with a level of material complexity confirmed by mass spectroscopy.

## Methods

### Preparation of collagen, fibrin, and humanized cell-free gels

Bovine collagen gels were prepared using liquid bovine type I collagen (5 mg mL^−1^, Symatese, F) and 10X MEM (Invitrogen, CH). The solution was neutralized with NaOH and set in a mold to create a bovine collagen gel. Three hundred microliter of fibrin precursor solutions containing 4 mg mL^−1^ of human fibrinogen (plasminogen, fibronectin-depleted; Enzyme Research Laboratories, South Bend, IN, USA), 2 U mL^−1^ factor XIIIa (Fibrogammin, CSL Behring, UK), 2 U mL^−1^ human thrombin (Sigma Aldrich, Switzerland), and 5 mM Ca^2+^ in tris-buffered saline (TBS) was cast into a mold. To ensure full fibrin polymerization, the gels were placed inside an incubator at 37 °C for 45 min. Compressed rat-tail collagen sheets laminated with fibrin (CFC gel) were prepared according to the same method as presented by Vardar et al.^31^, using 2.05 mg mL^−1^ rat-tail collagen type I (First-link, UK). For the preparation of cell-free humanized collagen grafts, 1 million primary human smooth muscle cells in cell culture media (alpha-MEM supplemented with 10% FBS, 1% L-glutamine, 1% Penicillin/Streptomycin and Fungizone) were added into each collagen solution instead of alpha-MEM as used for CFC gels prior to casting into the mold. Cell-seeded sheets were covered with cell culture medium and incubated at 37 °C with 5% CO_2_ for 2 or 4 weeks. The medium was changed twice a week. Decellularization of cell-seeded sheets was performed using a previously established protocol ensuring low dsDNA content^33^. Primary human bladder cells were isolated from donors after patient consent and ethical board approval from University Hospital of Lausanne (CHUV, Switzerland).

### Histology characterization

Biopsies were fixed in 4% PFA and embedded in paraffin. Sections were prepared by microtome cut at a thickness of 8 µm. Masson’s Trichrome staining of collagen and fibrin was performed according to provider instruction. Briefly, paraffin sections were dewaxed, hydrated, and treated in the Bouin’s solution at 60 °C for 1 h (750 ml of picric acid saturated in water, Sigma Aldrich; 250 ml of formaldehyde 37%, Acros; 50 ml of glacial acetic acid, Merck). Afterward, sections were washed with a DI water until disappearance of the yellow color, then incubated with Weigert’s iron hematoxylin for 5 min (10 g of hematoxylin, Sigma Aldrich; 950 ml of absolute ethanol mixed in 50 ml of MiliQ grade water; 20 g of ammonium iron(III) sulfate dodecahydrate, Acros; 16 ml of sulfuric acid 96%, Applichem, mixed in 1000 ml of MiliQ water), washed in water for 10 min, and transferred into 1% acetic acid solution for 2 min. Staining in fuchsin acid/Biebrich scarlet solution was done for 2 min (5 g of Biebrich scarlet 99%, Acros; 500 ml of MiliQ water; 1 g of fuchsin acid, Sigma Aldrich; 1 ml of glacial acetic acid, Merck; 100 ml of MiliQ water), and followed by 1% acetic acid solution treatment for 2 min. Samples were then transferred into 1:1 phosphotungstic/phosphomolybdic acids solution for 10 min (1000 ml of MiliQ water, 25 g of phosphomolybdic acid hydrate, Fluka; 25 g of phosphotungstic acid, Merck), then 1% acetic acid solution for 2 min, and 3% solution of aniline blue for 5 min (15 g of aniline blue, Sigma Aldrich; 10 ml of glacial acetic acid, Merck; 500 ml of MiliQ water). Finally, before placing a cover slip on the section, sample was washed in DI water, 1% acetic acid, again in DI water and then dehydrated. Imaging was performed with Olympus AX70 microscope.

### Mass spectroscopy

The bovine collagen and CFC gel networks were digested with RIPA buffer. The samples were run on a SDS-PAGE gel. SDS-PAGE gel lanes corresponding to the collagen molecular weight were sliced into pieces and proteins were reduced/alkylated before overnight Trypsin (Promega) digestion^47^. Resulting peptides were extracted and dried by vacuum centrifugation. Afterward, peptides were desalted on C18 StageTips^48^, and dried again by vacuum centrifugation prior to mass spectrometric analysis. For LC-MS/MS analysis, peptides were resuspended and separated by reversed-phase chromatography on a Dionex Ultimate 3000 RSLC nanoUPLC system in-line connected with an Orbitrap Q Exactive mass spectrometer (Thermo Fischer Scientific). Database search was performed using MS-Amanda^49^, and SEQUEST in Proteome Discoverer v.1.4 against a home-made database containing the Bos Taurus reference proteome (Uniprot release 2017_05), the rattus norvegicus reference proteome (Uniprot release 2017_05), and the human reference proteome (UniProt release 2017_05). All searches were performed with trypsin cleavage specificity, with up to two missed cleavages allowed and ion mass tolerance of 20 ppm for the precursor and 0.05 Da for the fragments. Carbamidomethylation was set as a fixed modification, whereas oxidation (M), acetylation (Protein N-term), phosphorylation (STY), pyroglutamic acid (peptide N-term Q) were considered as variable modifications. Data were further processed and inspected in the Scaffold 4 software (Proteome Software) to obtain emPAI values for each sample^32^ with the following criteria used in the analysis: 1% FDR at protein level, two unique peptides per protein group, 1% FDR at peptide level. Common false positives such as human keratins were excluded in the analysis. The open source DAVID online software (https://david.ncifcrf.gov/) was used to select proteins identified with the GO-terms for extracellular space and extracellular region^35,36^.

### Cathodoluminescence sample preparation

Samples were fixed in 4% PFA (Sigma Aldrich) and embedded in paraffin. Eighty-micrometer-thick cross-section cuts were prepared using a microtome and mounted on clean silicon substrates. This ensured that the top surface of each cross-section sample would be within the cathodoluminescence focal plane over a large FOV. Next, cross-sections were deparaffinized using a classical xylene procedure, critical point dried, and finally sputtered with a 4 nm thin Au–Pd layer (Quorum, Q 150 Sputter Coater; 0.8–0.2 Au–Pd ratio) to ensure electrical contact with the microscope ground to avoid surface charging. Thickness of the metal coating layer was set at 4 nm to minimize absorption and scattering of the intrinsic cathodoluminescence emission.

### Cathodoluminescence measurements

Samples were measured using an Attolight Rosa 4634 microscope, operating at 8 keV beam energy and 10 nA probe current. SEM data were obtained with 1 µs pixel dwell time. Cathodoluminescence measurements were performed at room temperature with 20 ms pixel integration times, using 150 grove mm^−1^ and 500 nm blaze dispersion grating. Cathodoluminescence emission was detected using an Andor Newton 920 silicone-based CCD camera, cooled down to −80 °C. Obtained cathodoluminescence data was analyzed using the Attolight spectroscopy package for MountainsMap v7.4 (Digital Surf), and OriginPro 8.0 (OriginLab). Careful choice of the electron beam conditions and 4 nm thin Au–Pd coating allowed a compromise between the signal SNR and degradation of the organic material when exposing it to the incident electron beam. Up to three cathodoluminescence scans of the same area could be performed before cathodoluminescence signal reduction was observed. Adapting the microscope detection optics to this specific application, would allow the use of lower probe currents or shorter integration times, thereby reducing the degradation effect.

### Reporting summary

Further information on experimental design is available in the Nature Research Reporting Summary linked to this article.

## Supplementary information

Supplementary Information

Reporting Summary

## Data Availability

All data generated or analysed during this study are included in this published article (and its supplementary information file). The mass-spectrometry proteomics data have been deposited to the ProteomeXchange Consortium via the PRIDE partner repository with the dataset identifier PXD012407 and 10.6019/PXD012407.
