# Supplementary material for: Quantitative intrinsic auto-cathodoluminescence can resolve spectral signatures of tissue-isolated collagen extracellular matrix
Source: Commun Biol. 2019 Feb 18;2:69. doi: 10.1038/s42003-019-0313-x (PMC6379429; doi:10.1038/s42003-019-0313-x)
Supplement: Supplementary file 1 — Supplementary Information [file 42003_2019_313_MOESM1_ESM.pdf]

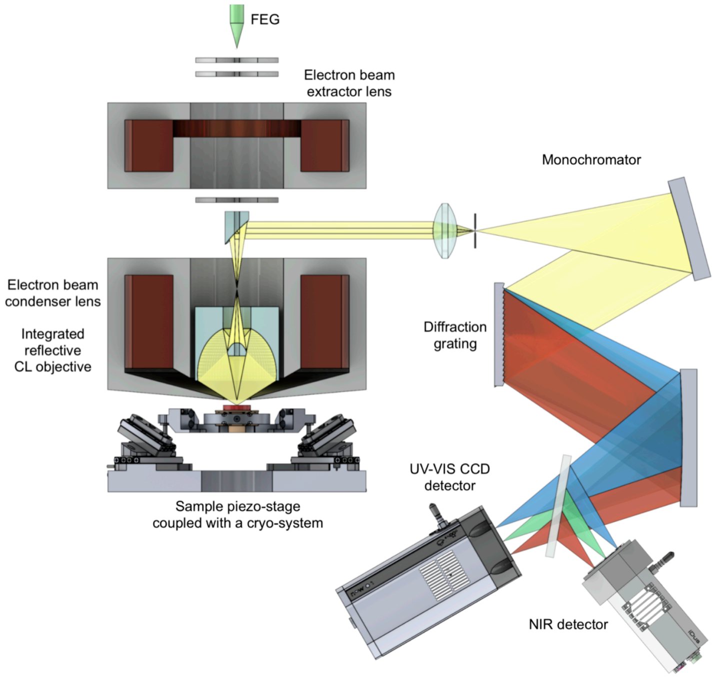

Supplementary Figure 1 | Schematic diagram of the quantitative Attolight Rosa 4634 CL-SEM microscope setup. An electron beam produced by a Schottky field emission gun (FEG) is accelerated by the extractor electromagnetic lens, passes through a hybrid optical objective system integrated within the focusing electromagnetic lens condenser, and irradiates the sample specimen loaded in a high vacuum chamber. This geometry assures the secondary electron (SE) contrast focal plane to be exactly matched with the optimal CL collection efficiency and allows for correlation of both image contrasts, recorded simultaneously pixel-by-pixel when the electron beam raster scans the sample. Emitted CL light is collected by the integrated reflective objective (numerical aperture NA = 0.71), collimated and directed into a monochromator to be dispersed on a reflective diffraction grating and finally spectrally resolved by the coupled detector, appropriate to a corresponding emission wavelength. The system operates in a continuous electron beam mode and allows for a broadband hyperspectral CL imaging from UV-VIS to NIR spectral range. Measurements can be performed at room and cryogenic temperature conditions (temperature range is 7-320 K).

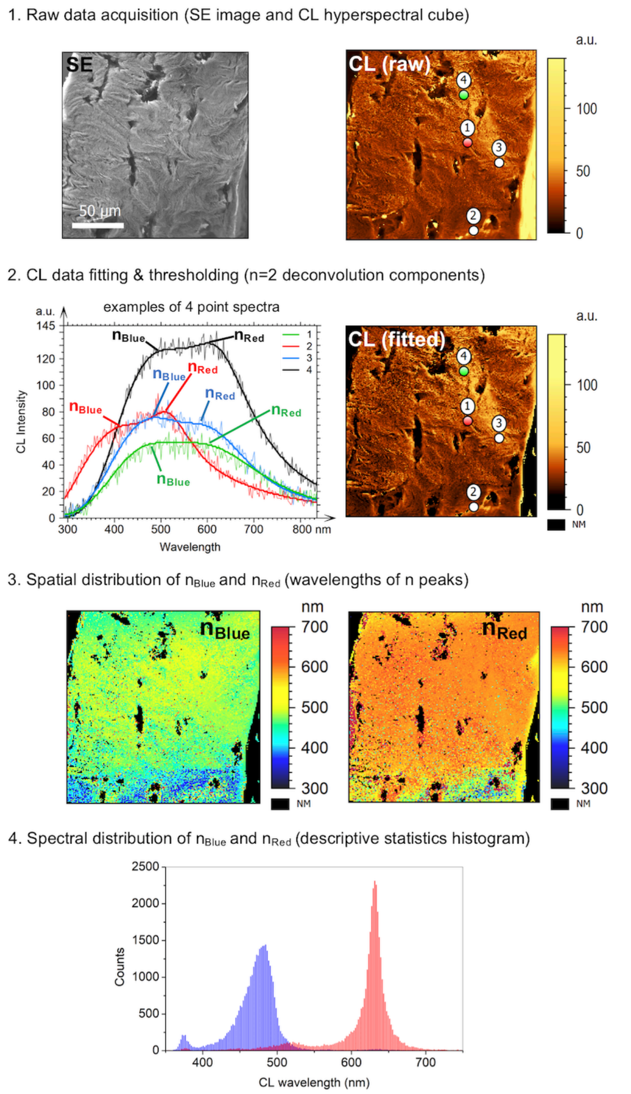

Supplementary Figure 2 | Step-by-step CL data processing method. Sample is a bovine collagen containing hSMC cells (before decellularization process). (**1**) Acquisition of the raw data set (SE image and CL hyperspectral cube) for the same FOV. (**2**) CL spectra fitting using a pseudo-Voigt function (one spectrum corresponds to one pixel in the CL hyperspectral image). Spectra below or above the intensity threshold range are being excluded (NM points). Deconvolution assumes two spectral components: n_{Blue} and n_{Red}, and CL peak wavelengths of each of these components are obtained. (**3**) Spatial distribution maps of n_{Blue} and n_{Red} peak wavelengths (color coded in nm). (**4**) Spectral distribution histogram of n_{Blue} and n_{Red} components (based on descriptive statistics). Final interpretation of the spectral distribution histogram requires comparison with the CL reference sample and correlation with a mass spec data.

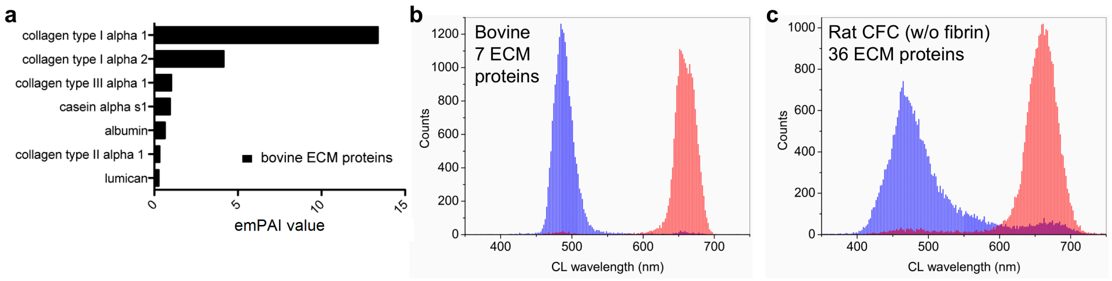

Supplementary Figure 3 | Mass spectra data of expressed ECM proteins in a bovine collagen gel and corresponding deconvoluted CL histogram. (**a**) Bovine collagen gel sample with 7 bovine proteins detected using DAVID online software using extracellular space as a selection GO-term. (**b**) Corresponding deconvoluted CL histogram, showing very narrow distributions of both spectral components, related to a low amount of detected bovine ECM proteins emitting with lower spectral dispersion. (**c**) Deconvoluted CL histogram of the rat CFC collagen (with subtracted fibrin signal) obtained from a gel with 36 ECM proteins detected by mass spectroscopy (Fig. 5a), presented here for comparison.

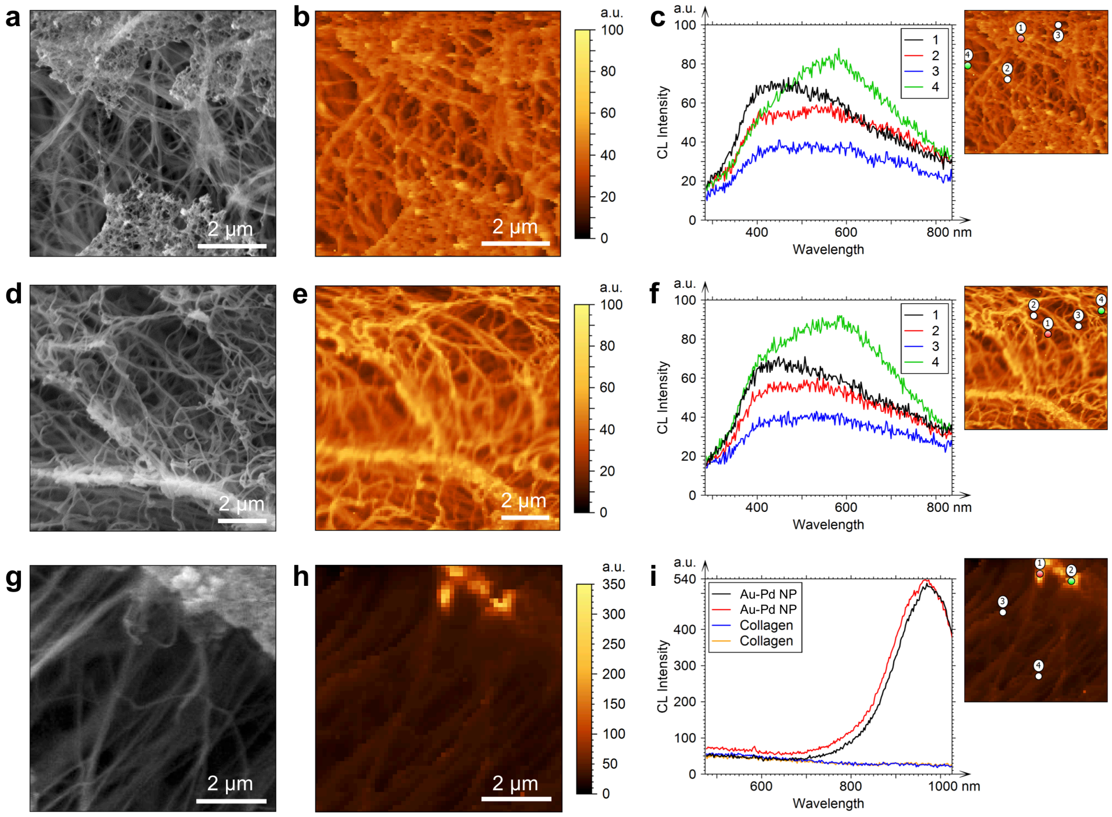

Supplementary Figure 4 | Comparison of CL signal from bovine collagen sputter-coated with 4 nm and 10 nm thick layers of Au-Pd. (**a**) SEM image of the 4 nm thick Au-Pd coated sample,

corresponding CL image (**b**) and point spectra from isolated fibrils (**c**). (**d**) SEM image of the 10 nm thick Au-Pd coated sample, corresponding CL image (**e**) and extracted point spectra from isolated fibrils (**f**). (**g**) SEM image of the 4 nm Au-Pd coated sample with large Au-Pd nanoparticles (NP), corresponding CL image (**b**) and extracted point spectra from NP's and isolated fibrils (**c**).

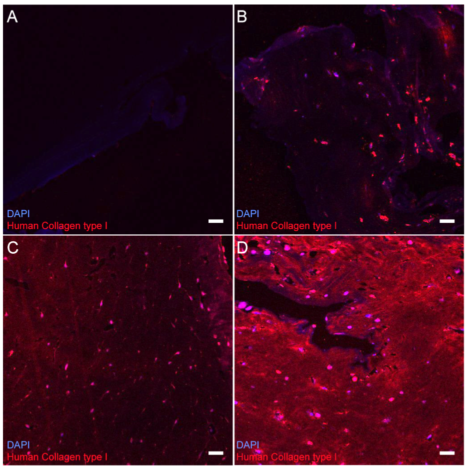

Supplementary Figure 5 | Fluorescence images of cell-free humanized-rat extracellular matrix grafts with human smooth muscle cells seeded inside CFC gels. Gradual turnover of CFC gels of rat to human extracellular matrix as identified by immunohistochemistry with a specific human collagen type I antibody. Cell nuclei were counterstained with DAPI. (**a**) Cell-free rat collagen sample. (**b**) 7 days culture of human smooth muscle cells inside CFC. (**c**) 14 days culture of human smooth muscle cells inside CFC. (**d**) 28 days culture of human smooth muscle cells inside CFC. Scale bars are 20 µm.

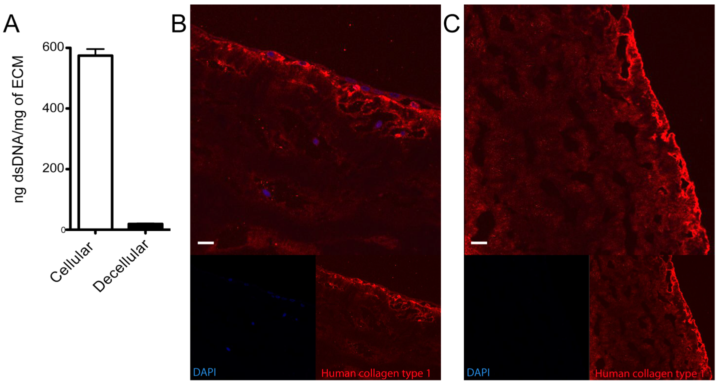

Supplementary Figure 6 | Analysis of decellularization treatment. hSMC were cultured during 4 weeks within CFC gels prior to decellularization. (**a**) Assessment of dsDNA content before and after decellularization treatment with Picogreen. (**b**) and (**c**) Immunohistochemistry to detect human specific

collagen type I within the graft before (**b**) and after the decellularization treatment (**c**). Scale bars are 20 µm.

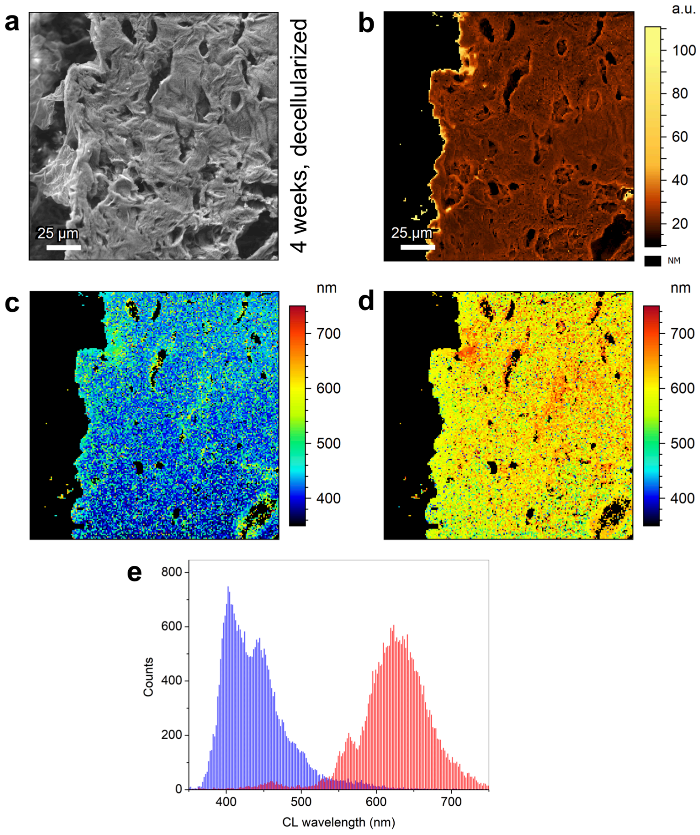

Supplementary Figure 7 | Additional CL data set for the 4 weeks hSMC cell-remodeled sample, obtained roughly 4 mm away from the scan in Fig. 5c. (**a**) SE image of the sample cross-section. (**b**) Corresponding CL image, and (**c,d**) spatial distributions of the two deconvoluted components – blue and red, respectively. (**e**) Spectral histogram of the two deconvoluted components.

Supplementary Tables

Supplementary Table 1 | All bovine proteins detected in bovine gel sample.

Identified Bovine Proteins (13)	Accession Number	emPAI Value
Uncharacterized protein OS=Bos taurus	F1N757	0.0059538
Collagen alpha-1(IV) chain OS=Bos taurus GN=COL4A1	G1K238	0.082339
Lumican OS=Bos taurus GN=LUM	Q05443	0.26232
Keratin, type II cuticular Hb3 OS=Bos taurus GN=KRT83	A4FUZ0	0.26609
Collagen alpha-1(II) chain OS=Bos taurus GN=COL2A1	P02459	0.31575
Serum albumin OS=Bos taurus	A0A140T897	0.62586

Hemoglobin fetal subunit beta OS=Bos taurus	P02081	0.89589
Alpha-S1-casein OS=Bos taurus	P02662	0.9345
Collagen alpha-1(III) chain OS=Bos taurus	F1MXS8	1.009
Actin, cytoplasmic 1 OS=Bos taurus	P60712	1.1626
Cytochrome c OS=Bos taurus	P62894	4.1331
Collagen alpha-2(I) chain OS=Bos taurus	P02465	4.1442
Collagen alpha-1(I) chain OS=Bos taurus	P02453	13.373

Supplementary Table 2 | All rat proteins detected in CFC gel sample.

Identified Rat Proteins (62)	Accession Number	emPAI Value
Olfactomedin-like protein 3 OS=Rattus norvegicus GN=Olfml3	B0BNI5	1.0823
Collagen alpha-1(XII) chain OS=Rattus norvegicus GN=Col12a1	D3Z9F8	1.5752
Protein Gsdma OS=Rattus norvegicus GN=Gsdma	D3ZA32	0.094265
Protein Angptl7 OS=Rattus norvegicus GN=Angptl7	D3ZDK4	0.61535
Cartilage intermediate layer protein 2 (Predicted) OS=Rattus norvegicus GN=Cilp2	D3ZE05	0.78957
Protein Col6a1 OS=Rattus norvegicus GN=Col6a1	D3ZUL3	1.3433
Osteoglycin (Predicted) OS=Rattus norvegicus GN=Ogn	D3ZVB7	3.6697
Keratocan (Predicted) OS=Rattus norvegicus GN=Kera	D3ZVD7	3.3482
Protein Col6a3 OS=Rattus norvegicus GN=Col6a3	D4A111	13.025
Aggrecan core protein OS=Rattus norvegicus GN=Acan	D4A7Y1	0.041617
Protein Tgfbi OS=Rattus norvegicus GN=Tgfbi	D4A8G5	3.0382
Protein Tnxb OS=Rattus norvegicus GN=Tnxb	E9PU73	2.8977
Protein LOC299282 OS=Rattus norvegicus GN=LOC299282	F1LM05	0.21558
Thrombospondin-4 (Fragment) OS=Rattus norvegicus GN=Thbs4	F1LMS5	0.6376
Procollagen, type VI, alpha 2, isoform CRA_a OS=Rattus norvegicus GN=Col6a2	F1LNH3	0.83737
Collagen alpha-1(XII) chain (Fragment) OS=Rattus norvegicus GN=Col12a1	F1LQC3	2.318
Lipid phosphate phosphatase-related protein type 2 OS=Rattus norvegicus GN=Prg4	F1LRA5	0.19855
Collagen alpha-1(II) chain OS=Rattus norvegicus GN=Col2a1	F1LRM7	0.41025
Protein Myh2 OS=Rattus norvegicus GN=Myh1	F1LRV9	0.22222
Collagen alpha-2(I) chain OS=Rattus norvegicus GN=Col1a2	F1LS40	12.69
Protein Abi3bp OS=Rattus norvegicus GN=Abi3bp	F1M9R3	0.35153
Fibromodulin OS=Rattus norvegicus GN=Fmod	G3V6E7	7.9143
Protein LOC100909710 OS=Rattus norvegicus GN=Myoc	G3V6E8	0.93893
Vimentin OS=Rattus norvegicus GN=Vim	G3V8C3	2.104
Fibrillin 1, isoform CRA_a OS=Rattus norvegicus	G3V9M6	0.012697

GN=Fbn1		
Protein Tnc OS=Rattus norvegicus GN=Tnc	M0RA80	0.69692
Cartilage oligomeric matrix protein OS=Rattus norvegicus GN=Comp	M0RBU0	0.80649
Chondroadherin OS=Rattus norvegicus GN=Chad	O70210	0.30557
Anionic trypsin-1 OS=Rattus norvegicus GN=Prss1	P00762	4.9931
Complement C3 OS=Rattus norvegicus GN=C3	P01026	0.21806
Hemoglobin subunit beta-1 OS=Rattus norvegicus GN=Hbb	P02091	0.41323
Collagen alpha-1(I) chain OS=Rattus norvegicus GN=Col1a1	P02454	22.649
Serum albumin OS=Rattus norvegicus GN=Alb	P02770	23.894
Fibronectin OS=Rattus norvegicus GN=Fn1	P04937	0.20401
Annexin A5 OS=Homo sapiens GN=ANXA5	P08758	0.13103
Serotransferrin OS=Rattus norvegicus GN=Tf	P12346	4.1506
Collagen alpha-1(III) chain OS=Rattus norvegicus GN=Col3a1	P13941	0.30389
Alpha-1-inhibitor 3 OS=Rattus norvegicus GN=A1i3	P14046	0.08944
Fibrinogen beta chain OS=Rattus norvegicus GN=Fgb	P14480	3.4529
Ubiquitin-60S ribosomal protein L40 OS=Rattus norvegicus GN=Uba52	P62986	0.32765
Actin, cytoplasmic 2 OS=Rattus norvegicus GN=Actg1	P63259	3.6638
Actin, alpha cardiac muscle 1 OS=Rattus norvegicus GN=Actc1	P68035	3.1487
Tubulin beta-5 chain OS=Rattus norvegicus GN=Tubb5	P69897	0.3231
Lactadherin OS=Rattus norvegicus GN=Mfge8	P70490	0.43894
Decorin OS=Rattus norvegicus GN=Dcn PE=1 SV=1	Q01129	17.767
Annexin A2 OS=Rattus norvegicus GN=Anxa2	Q07936	1.9233
EH domain-containing protein 2 OS=Rattus norvegicus GN=Ehd2	Q4V8H8	0.040466
Protein Serpinc1 OS=Rattus norvegicus GN=Serpinc1	Q5M7T5	0.089794
Alpha-1-macroglobulin OS=Rattus norvegicus GN=A1m	Q63041	0.039829
Keratin, type I cytoskeletal 19 OS=Rattus norvegicus GN=Krt19	Q63279	0.92076
Inter-alpha-trypsin inhibitor heavy chain H3 OS=Rattus norvegicus GN=Itih3	Q63416	0.84458
Olfactomedin-like protein 1 OS=Rattus norvegicus GN=Olfml1	Q66H86	0.68378
Gelsolin OS=Rattus norvegicus GN=Gsn	Q68FP1	0.34327
Tubulin alpha-1C chain OS=Rattus norvegicus GN=Tuba1c	Q6AYZ1	1.1316
Keratin, type I cytoskeletal 42 OS=Rattus norvegicus GN=Krt42	Q6IFU7	1.2682
Keratin, type I cytoskeletal 10 OS=Rattus norvegicus GN=Krt10	Q6IFW6	4.4795
Keratin, type II cytoskeletal 1 OS=Rattus norvegicus GN=Krt1	Q6IMF3	2.7904
Tubulin alpha-1B chain OS=Rattus norvegicus	Q6P9V9	1.1488

GN=Tuba1b		
Alpha-2 antiplasmin OS=Rattus norvegicus GN=Serpinf1	Q80ZA3	8.4915
Prolargin OS=Rattus norvegicus GN=Prelp	Q9EQP5	2.0673
Collagen alpha-1(V) chain OS=Rattus norvegicus GN=Col5a1	Q9JI03	0.12547
Guanine deaminase OS=Rattus norvegicus GN=Gda	Q9JKB7	0.25906

Supplementary Table 3 | All human and rat proteins detected in 4 weeks cell-free sample.

Identified Rat and human proteins (116)	Accession Number	emPAI Value
Tenascin-X OS=Homo sapiens GN=TNXB	E7EPZ9	0.008111
Protein Tnxb OS=Rattus norvegicus GN=Tnxb	E9PU73	0.029534
Cartilage oligomeric matrix protein OS=Rattus norvegicus GN=Comp	M0RBU0	0.045368
Collagen alpha-1(XVI) chain OS=Homo sapiens GN=COL16A1	Q07092	0.047772
Laminin subunit alpha-5 OS=Homo sapiens GN=LAMA5	O15230	0.056962
Isoform 2 of Collagen alpha-1(XVIII) chain OS=Homo sapiens GN=COL18A1	P39060-1	0.074167
Protein Abi3bp OS=Rattus norvegicus GN=Abi3bp	F1M9R3	0.075845
Collagen alpha-1(V) chain OS=Rattus norvegicus GN=Col5a1	Q9JI03	0.086367
Cartilage intermediate layer protein 2 (Predicted) OS=Rattus norvegicus GN=Cilp2	D3ZE05	0.089047
Pentraxin-related protein PTX3 OS=Homo sapiens GN=PTX3	P26022	0.089899
Collagen alpha-1(VII) chain OS=Homo sapiens GN=COL7A1	Q02388	0.091069
Alpha-1-macroglobulin OS=Rattus norvegicus GN=A1m	Q63041	0.091843
Chondroadherin OS=Rattus norvegicus GN=Chad	O70210	0.093415
Cell migration-inducing and hyaluronan-binding protein OS=Homo sapiens GN=CEMIP	Q8WUJ3	0.10069
Collagen alpha-1(XV) chain OS=Homo sapiens GN=COL15A1	P39059	0.10898
Laminin subunit beta-2 OS=Homo sapiens GN=LAMB2	P55268	0.11893
Glypican-6 OS=Homo sapiens GN=GPC6	Q9Y625	0.12276
Versican core protein OS=Homo sapiens GN=VCAN	P13611	0.14783
Protein Gsdma OS=Rattus norvegicus GN=Gsdma	D3ZA32	0.15995
Collagen alpha-1(XXVI) chain OS=Homo sapiens GN=COL26A1	Q96A83	0.17197
Laminin subunit alpha-4 OS=Homo sapiens GN=LAMA4	A0A0A0MQS9	0.17651
Syndecan-4 OS=Homo sapiens GN=SDC4	P31431	0.1769
Alpha-1-inhibitor 3 OS=Rattus norvegicus GN=A1i3	P14046	0.19095
Protein Angptl7 OS=Rattus norvegicus GN=Angptl7	D3ZDK4	0.20207
Collagen alpha-1(V) chain OS=Homo sapiens GN=COL5A1	A0A087WXW9	0.21707
Protein Myh2 OS=Rattus norvegicus GN=Myh1	F1LRV9	0.24765
Protein Serpinc1 OS=Rattus norvegicus GN=Serpinc1	Q5M7T5	0.25008
Destrin OS=Homo sapiens GN=DSTN	F6RFD5	0.25116

Olfactomedin-like protein 1 OS=Rattus norvegicus GN=Olfml1	Q66H86	0.26599
Lactadherin OS=Rattus norvegicus GN=Mfge8	P70490	0.27648
Inter-alpha-trypsin inhibitor heavy chain H3 OS=Rattus norvegicus GN=Itih3	Q63416	0.28553
EH domain-containing protein 2 OS=Rattus norvegicus GN=Ehd2	Q4V8H8	0.2973
Complement C3 OS=Rattus norvegicus GN=C3	P01026	0.30224
Protein Tnc OS=Rattus norvegicus GN=Tnc	M0RA80	0.32162
Thrombospondin-4 (Fragment) OS=Rattus norvegicus GN=Thbs4	F1LMS5	0.33056
Ferritin heavy chain OS=Homo sapiens GN=FTH1	P02794	0.38991
Extracellular matrix protein 1 OS=Homo sapiens GN=ECM1	Q16610	0.40679
Collagen alpha-1(II) chain OS=Rattus norvegicus GN=Col2a1	F1LRM7	0.42021
Laminin subunit beta-1 OS=Homo sapiens GN=LAMB1	G3XAI2	0.43245
Collagen alpha-1(III) chain OS=Rattus norvegicus GN=Col3a1	P13941	0.43954
Collagen alpha-1(III) chain OS=Homo sapiens GN=COL3A1	P02461	0.44065
Isoform 6 of Agrin OS=Homo sapiens GN=AGRN	O00468-6	0.44857
Nidogen-1 OS=Homo sapiens GN=NID1	P14543	0.4708
Collagen alpha-1(II) chain OS=Homo sapiens GN=COL2A1	P02458	0.48763
Protein LOC100909710 OS=Rattus norvegicus GN=Myoc	G3V6E8	0.51491
Fibrillin 1, isoform CRA_a OS=Rattus norvegicus GN=Fbn1	G3V9M6	0.57021
Coagulation factor XIII A chain OS=Homo sapiens GN=F13A1	P00488	0.57382
Serotransferrin OS=Rattus norvegicus GN=Tf	P12346	0.5856
Osteoglycin (Predicted) OS=Rattus norvegicus GN=Ogn	D3ZVB7	0.67413
Laminin subunit gamma-1 OS=Homo sapiens GN=LAMC1	P11047	0.69777
Fibulin-2 OS=Homo sapiens GN=FBLN2	P98095	0.71046
Vitronectin OS=Homo sapiens GN=VTN	P04004	0.72535
Procollagen-lysine,2-oxoglutarate 5-dioxygenase 1 OS=Homo sapiens GN=PLOD1	Q02809	0.836
Nidogen-2 OS=Homo sapiens GN=NID2	Q14112	0.86436
Keratin, type I cytoskeletal 19 OS=Rattus norvegicus GN=Krt19	Q63279	0.87791
Fibrillin-1 OS=Homo sapiens GN=FBN1	P35555	0.9205
Thrombospondin-2 OS=Homo sapiens GN=THBS2	P35442	0.93965
Olfactomedin-like protein 3 OS=Rattus norvegicus GN=Olfml3	B0BNI5	0.98785
Procollagen-lysine,2-oxoglutarate 5-dioxygenase 2 OS=Homo sapiens GN=PLOD2	O00469	1.1688
Lumican OS=Rattus norvegicus GN=Lum	P51886	1.1911
Keratocan (Predicted) OS=Rattus norvegicus GN=Kera	D3ZVD7	1.2588
Stromelysin-1 OS=Homo sapiens GN=MMP3	P08254	1.3103
Prolargin OS=Rattus norvegicus GN=Prelp	Q9EQP5	1.3389

Ig heavy chain V-III region GAL OS=Homo sapiens	P01781	1.4673
Serum albumin OS=Rattus norvegicus GN=Alb	P02770	1.7869
Basement membrane-specific heparan sulfate proteoglycan core protein OS=Homo sapiens GN=HSPG2	P98160	1.8713
Annexin A5 OS=Homo sapiens GN=ANXA5	P08758	1.9043
EMILIN-1 OS=Homo sapiens GN=EMILIN1	A0A0C4DFX3	2.4489
Fibrinogen alpha chain OS=Homo sapiens GN=FGA	P02671	2.5215
Collagen alpha-2(VI) chain OS=Homo sapiens GN=COL6A2	P12110	2.5934
Alpha-2 antiplasmin OS=Rattus norvegicus GN=Serpinf1	Q80ZA3	2.7205
Fibromodulin OS=Homo sapiens GN=FMOD	Q06828	2.7244
Annexin A2 OS=Rattus norvegicus GN=Anxa2	Q07936	3.5882
Collagen alpha-1(XII) chain OS=Rattus norvegicus GN=Col12a1	D3Z9F8	3.6254
Ubiquitin-60S ribosomal protein L40 OS=Rattus norvegicus GN=Uba52	P62986	3.6829
Procollagen, type VI, alpha 2, isoform CRA_a OS=Rattus norvegicus GN=Col6a2	F1LNH3	3.8923
Fibronectin OS=Rattus norvegicus GN=Fn1	P04937	4.0086
Collagen alpha-2(I) chain OS=Homo sapiens GN=COL1A2	A0A087WTA8	4.0119
Collagen alpha-1(XII) chain (Fragment) OS=Rattus norvegicus GN=Col12a1	F1LQC3	4.0377
Keratin, type I cytoskeletal 42 OS=Rattus norvegicus GN=Krt42	Q6IFU7	4.0911
Protein Tgfbi OS=Rattus norvegicus GN=Tgfbi	D4A8G5	4.3546
Isoform 3 of Periostin OS=Homo sapiens GN=POSTN	Q15063	4.689
Collagen alpha-1(VI) chain OS=Homo sapiens GN=COL6A1	A0A087X0S5	4.7968
Lumican OS=Homo sapiens GN=LUM	P51884	4.9599
Protein Col6a1 OS=Rattus norvegicus GN=Col6a1	D3ZUL3	5.0461
Anionic trypsin-1 OS=Rattus norvegicus GN=Prss1	P00762	5.1092
Hemoglobin subunit beta-1 OS=Rattus norvegicus GN=Hbb	P02091	5.2216
Protein Col6a3 OS=Rattus norvegicus GN=Col6a3	D4A111	5.4274
Dermcidin OS=Homo sapiens GN=DCD	P81605	5.4335
Isoform 4 of Tenascin OS=Homo sapiens GN=TNC	P24821-4	5.7395
Testican-1 OS=Homo sapiens GN=SPOCK1	Q08629	5.7877
Collagen alpha-1(XII) chain OS=Homo sapiens GN=COL12A1	D6RGG3	6.2087
Decorin OS=Rattus norvegicus GN=Dcn	Q01129	6.2094
Keratin, type II cytoskeletal 1 OS=Rattus norvegicus GN=Krt1	Q6IMF3	6.7336
Fibromodulin OS=Rattus norvegicus GN=Fmod	G3V6E7	6.846
Collagen alpha-3(VI) chain OS=Homo sapiens GN=COL6A3	P12111	7.9612
Tubulin beta-5 chain OS=Rattus norvegicus GN=Tubb5	P69897	8.0588
Serum albumin OS=Homo sapiens GN=ALB	P02768	9.6194
Collagen alpha-1(I) chain OS=Homo sapiens GN=COL1A1	P02452	9.8244

Tubulin alpha-1C chain OS=Rattus norvegicus GN=Tuba1c	Q6AYZ1	10.198
Biglycan OS=Homo sapiens GN=BGN	P21810	10.375
Galectin-1 OS=Homo sapiens GN=LGALS1	P09382	10.537
Decorin OS=Homo sapiens GN=DCN	P07585	10.897
Collagen alpha-2(I) chain OS=Rattus norvegicus GN=Col1a2	F1LS40	10.909
Myosin-9 OS=Homo sapiens GN=MYH9	P35579	11.66
Actin, alpha cardiac muscle 1 OS=Rattus norvegicus GN=Actc1	P68035	11.92
Thrombospondin-1 OS=Homo sapiens GN=THBS1	P07996	12.932
Tubulin alpha-1B chain OS=Rattus norvegicus GN=Tuba1b	Q6P9V9	12.947
Fibrinogen beta chain OS=Homo sapiens GN=FGB	P02675	13.417
Transforming growth factor-beta-induced protein ig-h3 OS=Homo sapiens GN=TGFBI	Q15582	16.213
Isoform 15 of Fibronectin OS=Homo sapiens GN=FN1	P02751-15	16.74
Vimentin OS=Homo sapiens GN=VIM	P08670	17.534
Keratin, type I cytoskeletal 10 OS=Rattus norvegicus GN=Krt10	Q6IFW6	17.647
Fibronectin OS=Homo sapiens GN=FN1	P02751	18.462
Fibrinogen gamma chain OS=Homo sapiens GN=FGG	P02679	18.923
Collagen alpha-1(I) chain OS=Rattus norvegicus GN=Col1a1	P02454	22.276
