# Supplementary material for: Quantitative intrinsic auto-cathodoluminescence can resolve spectral signatures of tissue-isolated collagen extracellular matrix
Source: Commun Biol. 2019 Feb 18;2:69. doi: 10.1038/s42003-019-0313-x (PMC6379429; doi:10.1038/s42003-019-0313-x)
Supplement: Supplementary file 2 — Reporting Summary [file 42003_2019_313_MOESM2_ESM.pdf]

nature research

Corresponding author(s): Hans Mattias Larsson

Reporting Summary

Nature Research wishes to improve the reproducibility of the work that we publish. This form provides structure for consistency and transparency in reporting. For further information on Nature Research policies, see Authors & Referees and the Editorial Policy Checklist.

Statistical parameters

When statistical analyses are reported, confirm that the following items are present in the relevant location (e.g. figure legend, table legend, main text, or Methods section).

n/a	Confirmed	
☐	☒	The exact sample size (n) for each experimental group/condition, given as a discrete number and unit of measurement
☐	☒	An indication of whether measurements were taken from distinct samples or whether the same sample was measured repeatedly
☐	☒	The statistical test(s) used AND whether they are one- or two-sided<br>*Only common tests should be described solely by name; describe more complex techniques in the Methods section.*
☐	☒	A description of all covariates tested
☒	☐	A description of any assumptions or corrections, such as tests of normality and adjustment for multiple comparisons
☐	☒	A full description of the statistics including central tendency (e.g. means) or other basic estimates (e.g. regression coefficient) AND variation (e.g. standard deviation) or associated estimates of uncertainty (e.g. confidence intervals)
☒	☐	For null hypothesis testing, the test statistic (e.g. F, t, r) with confidence intervals, effect sizes, degrees of freedom and P value noted<br>*Give P values as exact values whenever suitable.*
☒	☐	For Bayesian analysis, information on the choice of priors and Markov chain Monte Carlo settings
☒	☐	For hierarchical and complex designs, identification of the appropriate level for tests and full reporting of outcomes
☒	☐	Estimates of effect sizes (e.g. Cohen's d, Pearson's r), indicating how they were calculated
☐	☒	Clearly defined error bars<br>*State explicitly what error bars represent (e.g. SD, SE, CI)*

Our web collection on statistics for biologists may be useful.

Software and code

Policy information about availability of computer code

Data collection	CL and SE data was obtained using Attolight Rosa 4634 CL-SEM microscope. For LC MS/MS analysis, peptides were resuspended and separated by reversed-phase chromatography on a Dionex Ultimate 3000 RSLC nanoUPLC system in-line connected with an Orbitrap Q Exactive mass spectrometer (Thermo Fischer Scientific). Fluorescence data was collected using Zeiss LSM 700 laser scanning confocal microscope. The Masson’s trichrome data was obtained using Olympus AX70 microscope.
Data analysis	CL/SEM sata analysis was performed using Digital Surf MountainsMap software with Attolight CL spectroscopy package (v7.4). Spectral histograms were plotted and analyzed using OriginPro 8.0 (from OriginLab). The mass spec data was processed and inspected with the Scaffold 4 software (Proteome Software) to obtain emPAI values for each sample, and finally plotted using Prism 4 software. The open source DAVID online software (https://david.ncifcrf.gov/) was used to select proteins identified with the GO-terms for extracellular space and extracellular region.

For manuscripts utilizing custom algorithms or software that are central to the research but not yet described in published literature, software must be made available to editors/reviewers upon request. We strongly encourage code deposition in a community repository (e.g. GitHub). See the Nature Research guidelines for submitting code & software for further information.

Data

Policy information about availability of data

All manuscripts must include a data availability statement. This statement should provide the following information, where applicable:
- Accession codes, unique identifiers, or web links for publicly available datasets
- A list of figures that have associated raw data
- A description of any restrictions on data availability

The data that support the plots within this manuscript and other findings of this study are available from corresponding author upon reasonable request.

Field-specific reporting

Please select the best fit for your research. If you are not sure, read the appropriate sections before making your selection.

☒ Life sciences ☐ Behavioural & social sciences ☐ Ecological, evolutionary & environmental sciences

For a reference copy of the document with all sections, see nature.com/authors/policies/ReportingSummary-flat.pdf

Life sciences study design

All studies must disclose on these points even when the disclosure is negative.

Sample size	No sample-size calculation was performed for the CL-SEM study.
Data exclusions	In the hyperspectral CL data scans, all the measurement points with an intensity below 10 counts (recorded over 20 ms integration times) were excluded to avoid false peak fitting and deconvolution results. These points resulted from the CCD detector dark noise, and were marked as 'NM' (non-measurable points). Presented spectral histograms do not contain these 'NM' data points.
Replication	Each presented sample was CL-probed 40'000 times to obtain a data set over a whole cross-section area. Each sample type (CFC, bovine, fibrin, humanized scaffolds) was scanned at three different areas showing very similar spectral responses for particular samples. However, the heterogeneity of these samples prevents direct comparison side-by-side. The study has been designed that way, to demonstrate a capability of the CL technique in characterization of biomaterials, and not to provide an absolute information about the studied materials - this is the scope of another manuscript in preparation.
Randomization	Each sample type was selected with an increase in molecular heterogeneity. To demonstrate the CL-technique capacity in molecular identification, we have firstly scanned a reference fibrin and bovine/rat collagen samples, and further performed characterization of CFC and human-cell remodeled CFC samples after 2 and 4 weeks of remodeling. The three different areas per sample in CL characterization were selected randomly on corresponding cross-sections.
Blinding	Sample bio-engineering process and preparation for CL imaging were performed by HML and MSZ was blinded during the CL imaging.

Reporting for specific materials, systems and methods

Materials & experimental systems

n/a	Involved in the study
☐	☒ Unique biological materials
☐	☒ Antibodies
☐	☒ Eukaryotic cell lines
☒	☐ Palaeontology
☒	☐ Animals and other organisms
☒	☐ Human research participants

Methods

n/a	Involved in the study
☒	☐ ChIP-seq
☒	☐ Flow cytometry
☒	☐ MRI-based neuroimaging

Unique biological materials

Policy information about availability of materials

Obtaining unique materials	Collagen and fibrin components are available from suppliers. Primary human bladder cells were isolated from donors after patient consent and ethical board approval from University hospital of Lausanne (CHUV, Switzerland).

Antibodies

Antibodies used	A collagen type I antibody from Abcam (ab34710) was used in the fluorescence study presented in the supplementary information file.
Validation	Provided on the Abcam website: https://www.abcam.com/collagen-i-antibody-ab34710.html

Eukaryotic cell lines

Policy information about cell lines

Cell line source(s)	Human cells isolated from bladder tissue.
Authentication	None of the cells were authenticated externally, the cells were isolated from primary source and characterized in-house (J Tissue Eng Regen Med. 2010 Feb;4(2):123-30. doi: 10.1002/term.222).
Mycoplasma contamination	The cells were not tested for mycoplasma.
Commonly misidentified lines (See ICLAC register)	Cells used in this manuscript were isolated primary human bladder smooth muscle cells, no misidentified line is applicable
